# The Effect of Zeolite Morphology and Loading on the Local Segmental Dynamics and Crystallisation Behaviour of PDMS–Zeolite Composites

**DOI:** 10.3390/polym17212911

**Published:** 2025-10-31

**Authors:** Tatjana Antonić Jelić, Damir Klepac, Leana Vratović, Dalibor Merunka, Jurica Jurec, Marin Tota, Kata Galić, Srećko Valić

**Affiliations:** 1 Ruđer Bošković Institute, Bijenička Cesta 54, 10000 Zagreb, Croatia; merunka@irb.hr (D.M.); jjurec@irb.hr (J.J.); 2 Faculty of Medicine, University of Rijeka, Braće Branchetta 20, 51000 Rijeka, Croatia; damir.klepac@uniri.hr (D.K.); vratovic@imc.sas.cz (L.V.); marin.tota@uniri.hr (M.T.); 3 Centre for Micro and Nano Sciences and Technologies, University of Rijeka, Radmile Matejčić 2, 51000 Rijeka, Croatia; 4 Faculty of Food Technology and Biotechnology, University of Zagreb, Pierottijeva 6, 10000 Zagreb, Croatia; kgalic@pbf.hr

**Keywords:** polydimethylsiloxane, zeolite, segmental dynamics, electron spin resonance (ESR), spin probe, crystallisation, DSC

## Abstract

The local segmental mobility of polymer chains in polydimethylsiloxane (PDMS) plays a critical role in determining the material’s behaviour. Incorporation of zeolite particles can modify these local dynamics, which is crucial as they affect the overall performance of the resulting composite material with potential for various industrial applications. The aim of this study was to investigate the influence of zeolite addition on the local dynamic behaviour of PDMS chain segments in PDMS–zeolite composites. To investigate the effect of zeolite morphology and loading on the segmental dynamics and phase behaviour of PDMS, Zeolite A (with cubic and spherical morphologies) and Zeolite X were incorporated into the PDMS matrix at 20, 30, and 40 wt%. The electron spin resonance (ESR)-spin probe method was used to study molecular dynamics, while the thermal behaviour was analysed using differential scanning calorimetry (DSC). ESR results revealed that the presence of zeolites increases the isothermal crystallisation rate affecting segmental mobility in the amorphous phase below the crystallisation temperature. This effect was found to depend more strongly on zeolite morphology than on filler content. DSC measurements showed no change in glass transition temperature with the addition of zeolite; however, shifts in cold crystallisation and melting behaviour were observed, indicating changes in crystal structure and its degree of perfection. These findings suggest that zeolites act as heterogeneous nucleation agents, with their structural properties playing a critical role in the crystallisation behaviour of PDMS.

## 1. Introduction

Polydimethylsiloxane (PDMS) has a low glass transition temperature and the ability to crystallise. For decades, it has attracted considerable scientific attention and remains an active subject of research. Although it was already known that PDMS could crystallise [1], its first crystalline structure was proposed by Damaschun in 1962 [2]. Despite numerous studies conducted over more than six decades [3,4,5,6,7,8,9,10,11,12], the complex structure of its crystalline phase has not been fully elucidated, and many questions remain unanswered [13]. Although the crystalline phase has been extensively studied, the dynamics of the amorphous chain segments in a semicrystalline PDMS matrix have received comparatively less attention. Massa et al. studied the dynamic behaviour in semicrystalline PDMS matrices using high-field ESR spectroscopy [14,15,16]. They confirmed the presence of two distinct amorphous phases in the interfacial region: the rigid amorphous fraction (RAF), a constrained environment located near the crystalline domains, and the mobile amorphous fraction (MAF), situated between the crystalline regions and exhibiting properties similar to those of a fully amorphous bulk polymer.

A simplified overview of the effects of cooling rate, polymer molecular weight, filler addition, and crosslinking on the low-temperature behaviour of PDMS, with particular emphasis on melting behaviour, is available in the literature [7]. It is known that the pure long-chain sample crystallises more rapidly than the short-chain sample. However, adding a certain number of short chains to a long-chain matrix can increase the crystallisation rate [6]. Differential scanning calorimetry (DSC) measurements with various additives indicate that the presence of nanometric solid particles also increases the crystallisation rate compared to pristine PDMS melts. This effect is attributed to entropic interactions in the boundary layer [8]. The results for PDMS–silica composites show that silica particles do not affect the size of crystallites but reduce the extent of crystallisation. Additionally, they exert two opposing effects on the crystallisation rate: at low silica volume fractions, nucleation dominates and promotes faster crystal growth, whereas at high silica fractions, topological constraints prevail and hinder crystal growth [9]. Another study reports that silica nanoclusters promote crystallisation, as evidenced by higher crystallisation temperatures upon cooling and lower crystallisation temperatures upon heating [11]. Both melt and glass crystallisations are significantly promoted in the presence of silica nanoclusters, with more pronounced secondary crystallisation and greater crystal perfection attributed to the structuring effect of the silica nanoclusters [11]. Fragiadakis et al. investigated the effects of nanoparticles on chain dynamics using a thermally stimulated depolarisation current, broadband dielectric relaxation spectroscopy, and differential scanning calorimetry [17]. They observed a slower relaxation of polymer chain segments near the polymer–filler interface, where mobility is restricted due to interactions with the filler surface. In contrast to the findings of other authors, their DSC analysis showed a change in the shape of the glass transition, along with a decrease in both the degree of crystallinity and the crystallisation rate upon the addition of silica.

Zeolites are often used as fillers in various types of PDMS-based membranes [10,18,19,20]. They are known to interact strongly with polymer matrices, potentially forming physical crosslinks via van der Waals forces or chemical crosslinks through reactions of surface hydroxyl groups with polymer chains. In PDMS, physical interactions, mainly the penetration of PDMS chains into zeolite pores, predominate. An investigation of PDMS–NaA zeolite composites reveals that zeolite addition alters the equilibrium swelling degree, hardness, and thermal stability, indicating that this type of zeolite acts as a reinforcing filler [18]. Significant changes in the degree of swelling and hardness were observed for zeolite concentrations lower than 20%, with a less pronounced effect at higher concentrations. A new bonding mechanism of zeolite within the composite was also proposed. A study of PDMS–NaY zeolite composite films showed that zeolite addition significantly improves mechanical and thermal properties [19]. Yang et al. reported that the incorporation of zeolites into PDMS membranes induces a crosslinking effect, leading to slightly enhanced selectivity and reduced flux in comparison to pristine PDMS membranes [20].

Using electron spin resonance (ESR) to investigate segmental dynamics in polymer-filler composites is a well-established approach in polymer science and materials chemistry. ESR is highly sensitive to molecular motion on the timescale from pico- to microseconds and can provide detailed insights into the local segmental mobility, matrix (in)homogeneity, and interactions at interfaces between the polymer and the filler [21,22,23,24]. Knowledge of segmental dynamics is important because it is directly linked to the mechanical (flexibility, strength, and elasticity), transport (gas permeability and separation), and interfacial properties (adhesion, stress transfer, and compatibility) between PDMS and zeolite surfaces of the composites. Understanding and controlling segmental dynamics is critical for optimising the performance of PDMS–fillers composites in applications such as gas separation membranes, flexible electronics, sensors, catalytic systems, biomedicine, etc. [25,26,27,28].

In this study, the electron spin resonance (ESR)-spin probe method, known as a powerful technique for investigating local molecular motions [21,22,23], was employed to study the segmental dynamics in PDMS–zeolite composites. The spin probe technique utilises free nitroxide radicals, which are mechanically embedded in the polymer matrix and sensitive to their local environment, enabling the characterisation of polymer chain mobility at the segmental level. It is known that the crystal shape of a zeolite (e.g., cubic, spherical, pyramidal) significantly affects its physicochemical properties, such as surface area, diffusion capacity, and pore distribution, which directly determine its performance in specific applications. Therefore, three types of zeolites with different structural characteristics and loadings were selected and incorporated into a model PDMS melt matrix to investigate the influence of filler morphology and content on local segmental dynamics.

The matrix consisted of uncrosslinked PDMS chains, allowing free movement of polymer segments and enabling clearer observation of how zeolite particles affect local dynamics. By analysing changes in ESR spectral parameters, such as correlation times and line shapes, insights were gained into how the presence of zeolites alters the segmental mobility of PDMS chains, reflecting interactions between the polymer and the filler surfaces.

## 2. Materials and Methods

### 2.1. Sample Preparation

Methyl-terminated polydimethylsiloxane (PDMS), *M*_w_ = 28,000, density 0.971 kg/L, was purchased from ThermoFisher (Kandel) GmbH (Kandel, Germany), and the free nitroxide radical used as a spin probe for ESR measurements, 4-oxo-2,2,6,6-tetramethyl-1-piperidinyloxy (4-oxo-TEMPO or TEMPONE), was obtained from Eastman Kodak Co., Rochester, NY, USA. Zeolite A with cubic and spherical morphologies was synthesised, while Zeolite X was purchased from Silkem d.o.o. For the synthesis of cubic Zeolite A, an aluminosilicate hydrogel with the molar oxide composition 4.72 Na_2_O × Al_2_O_3_ × 1.93 SiO_2_ × 254.86 H_2_O was prepared by mixing sodium silicate and sodium aluminate solutions of appropriate concentrations (in terms of Na_2_O and Al_2_O_3_) under continuous stirring with a magnetic stirrer. The sodium aluminate solution was prepared by dissolving anhydrous NaAlO_2_ (Riedel de Haen; 41 wt% Na_2_O, 54 wt% Al_2_O_3_, and 5 wt% H_2_O), while the sodium silicate solution was obtained by dissolving Na_2_SiO_3_ (Aldrich; 51 wt% Na_2_O, 48 wt% SiO_2_, and 1 wt% H_2_O) in demineralised water. The solutions were maintained at 25 °C prior to mixing. The prepared hydrogel was stirred for 10 min, then transferred to a preheated stainless-steel reaction vessel at the crystallisation temperature (80 °C) and heated for 4 h until crystallisation was complete, i.e., until the entire amorphous phase was fully converted into the crystalline phase. The reaction vessel was equipped with a thermostatic jacket with circulating water. The resulting product, Zeolite A, was washed to a pH of approximately 9 and dried at 105 °C for 24 h. A detailed procedure for the synthesis of spherical Zeolite A particles is provided in a patent [29] and the supporting literature [30,31], while the synthesis of cubic particles is described in reference [32]. The morphological forms of the zeolites used in this study are shown in Figure S1 in the Supplementary Materials.

The desired amounts of zeolites were incorporated into the PDMS matrix by directly mixing the zeolite with PDMS on a magnetic stirrer for 10 min at room temperature. The mixtures were then placed in an ultrasonic bath and sonicated for 15 min to achieve a homogeneous dispersion of zeolite particles within the PDMS matrix. The zeolites used in this study, as previously mentioned, were Zeolite A with cubic (AC) and spherical (AS) morphologies and Zeolite X (X) with pyramidal morphology. All composite samples were prepared using three loadings for each zeolite type. The loadings, expressed as mass percentages, were 20%, 30%, and 40%. The spin probe was incorporated into the PDMS–zeolite composites by mixing the nitroxide directly with samples on a magnetic stirrer for 10 min. The final probe concentration was approximately 0.05 wt%. The composition and labels of the samples investigated are presented in Table 1.

### 2.2. Electron Spin Resonance (ESR) Measurements

ESR measurements were performed using a Bruker EMX spectrometer operating at 9.3 GHz, equipped with a Bruker ER 041 XG microwave bridge and a Bruker ER 4111 VT temperature control unit, with an uncertainty of ±1 °C. To ensure uniform distribution of zeolite particles within the PDMS matrix, all samples were remixed and sonicated for 15 min before being placed in ESR tubes. The sample mass in the ESR tubes was approximately 30 mg. Initial measurements on pristine PDMS were carried out over a temperature range from −107 °C to 25 °C, with steps of 5 °C or 10 °C depending on the sensitivity of spectral line shape changes within specific temperature intervals. Prior to the first measurement, samples were quenched and held at −107 °C for 5 min. All subsequent spectra were recorded by gradually increasing the temperature, holding each sample at the target temperature for 2 min before data acquisition began.

The temperature of −85 °C, at which composite ESR spectra with two distinct components were observed, was selected as the optimal temperature for characterising the local motional behaviour of PDMS chain segments. All samples were subsequently measured isothermally at this temperature as a function of time. It should be noted that the samples were cooled directly from room temperature to −85 °C within the ESR cavity. Spectra were recorded every minute, starting from the first minute until no further changes in spectral shape or line intensity were detected. The spectral parameters were as follows: frequency 9.30 GHz, sweep width 100 G, sweep time 30.72 s, power 2.00 mW, modulation frequency 100 kHz, modulation amplitude 1.00 G, time constant 1.28 ms, and receiver gain 1·10^2^. Spectra acquisition and analysis were performed using WinEPR acquisition software (version 4.55). The ESR spectra were simulated using the spectral fitting programme NLSL (version 2004), which is based on the Stochastic Liouville Equation (SLE) [33]. The spin probe motion was assumed to follow the Brownian diffusion model. The **g** and **A** tensors were as follows: **g** = [2.0085, 2.0059, 2.0021] and **A** = [6.57, 6.46, 31.49]. Simulations were performed by using either one or two components. The fits were obtained by varying the isotropic Gaussian line broadening (*gib*0) and the rotational diffusion (*R*) for each component. The quality of the fit was determined using the correlation coefficient (*r*) which was greater than 0.98 for most fits. Rotational correlation times (*τ*_R_) were calculated according to the following equation:

*τ*_R_ = 1/6*R* (1)

### 2.3. Differential Scanning Calorimetry (DSC) Measurements

DSC measurements were carried out using a Mettler Toledo (Columbus, OH, USA) 822e DSC analyser over a temperature range from −150 °C to 25 °C. Samples were cooled from room temperature to −150 °C using the calorimeter’s uncontrolled cooling mode, with a maximum cooling rate of approximately 40 °C/min. Prior to measurement, the samples were held at −150 °C for 5 min before the temperature was increased at a rate of 10 °C/min. All measurements were performed in a nitrogen atmosphere with a flow rate of 20 mL/min. A sample mass of approximately 10 mg was placed in an aluminium DSC pan, which was subsequently sealed using a press. Data analysis was performed on a PC with STARe Software (v16.20). The degree of crystallinity was calculated using the theoretical enthalpy value Δ*H*_th_ = 37.4 J/g as given in reference [7]. Measured enthalpy values were corrected to account for the zeolite content in each sample.

To ensure sample homogeneity, minimise potential zeolite agglomeration, and achieve reproducible results, all ESR and DSC measurements were preceded by careful sample preparation. Two measurements were performed for each sample type. As indicated above, the samples were thoroughly homogenised prior to the measurements, and sub-samples were taken after repeated homogenisation. The results showed negligible variation between repeated measurements, indicating good reproducibility and confirming the representativeness of the investigated samples.

## 3. Results and Discussion

### 3.1. ESR Analysis

**Figure 1:**
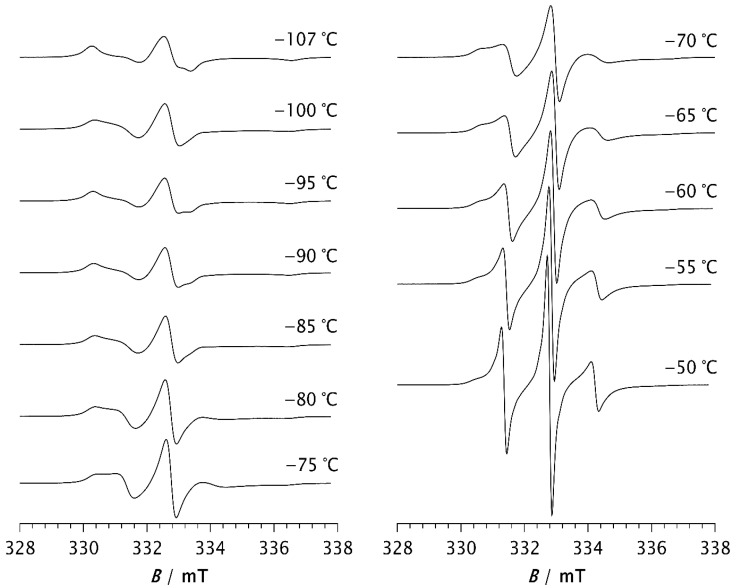
ESR spectra of the spin probe in PDMS measured at low temperature, i.e., between the glass transition temperature and the melting temperature.

**Figure 2:**
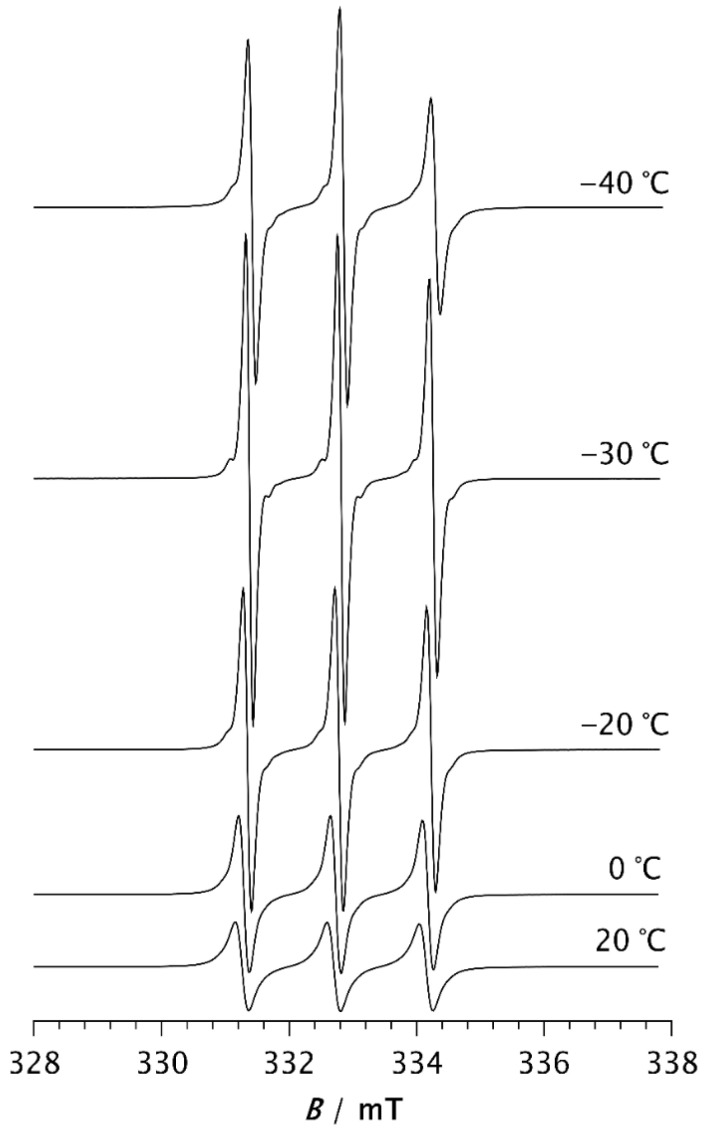
ESR spectra of the spin probe in PDMS measured above the melting temperature.

**Figure 3:**
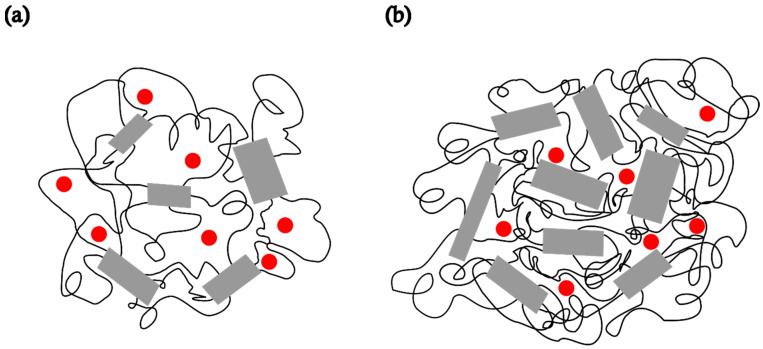
Schematic representation of a semicrystalline PDMS matrix and the distribution of the spin probe (●) inside the amorphous region of PDMS at −85 °C (**a**) after 2 min and (**b**) upon reaching steady state.

The ESR spectra of the spin probe in pristine PDMS, measured as a function of temperature, are shown in Figure 1 and Figure 2. Figure 1 shows the spectra recorded at low temperatures, between the glass transition (*T*_g_) and the melting point. At these temperatures, the spectra consist of three overlapping hyperfine lines, with a pronounced dominance of the broad component, characteristic of the slow spin probe motion [22,23]. The dynamics of the spin probe in a polymer matrix are generally influenced not only by the size of the pores in which the probe molecules reside but also by the local mobility of the polymer chain segments. As long as the matrix remains frozen (i.e., below *T*_g_), the motion of the spin probe is determined by the size of the pores. Additionally, the mobility of end and side-chain groups (if present) may contribute to the probe’s dynamics. At higher temperatures, the influence of local chain segmental mobility becomes dominant, causing the probe molecules to follow these segmental motions, which results in the narrowing of the spectral lines, as shown in Figure 2. The probe dynamics observed at low temperatures, Figure 1, reflect the dynamic behaviour of chain segments in the amorphous phase of the semicrystalline sample. According to the DSC results reported below, as well as the literature data [3,4,11,34], a portion of the PDMS chains forms a crystalline phase, while the rest remains amorphous. Our previous study [35] demonstrated that the probe molecules are excluded from the crystalline phase and diffuse only through the amorphous phase. The spin probe distribution inside semicrystalline PDMS at the beginning of the crystallisation and after its completion is shown schematically in Figure 3a and Figure 3b, respectively.

Spectra observed at temperatures between −107 °C and −80 °C show a slight change in spectral line shape, indicating the appearance of a narrow component with increasing temperature which becomes more noticeable for *T* > −75 °C. The increase in the narrow component, attributed to fast probe motion, is accompanied by a decrease in the broad component, which reflects slow probe motion. Such composite spectra, typically observed in dynamically inhomogeneous systems, indicate the coexistence of well-resolved slow and fast local segmental motions [22,23,24]. Based on previous studies of semicrystalline PDMS [15,16], probe molecules with fast and intermediate mobility are located in the disordered fraction far from the crystallites (MAF), while those with extremely low mobility are trapped near to the crystallites in a glassy environment (RAF) that persists up to the melting of PDMS. The increase in fast molecular motions with increasing temperature is therefore attributed to the growth of the MAF fraction at the expense of the RAF. In other words, segments located in the middle of the amorphous phase move faster and contribute to the narrow component in the ESR spectrum, whereas segments emerging from, or located near, the crystalline phase move more slowly and contribute to the broad component.

At −50 °C, the fast component becomes very pronounced. As the temperature increases further, the slow component disappears, so that the spectrum at −30 °C consists of three well-resolved narrow lines, as seen in Figure 2. This change can be associated with the melting of the crystalline phase and its transition to the amorphous phase (see thermal analysis results below). At temperatures above −30 °C, the line width increases with increasing temperature. It is known that at short correlation times, as long as *τ*_R_ < 0.02 ns, the contribution of spin rotation to the transverse relaxation rate (*T*_2_^−1^) dominates. At longer *τ*_R_ values this contribution becomes less significant, and the rotational modulation of the ^14^N hyperfine and **g** tensors anisotropies begins to dominate [36]. This effect becomes increasingly pronounced with rising temperature up to 20 °C, as seen in Figure 2.

**Figure 4:**
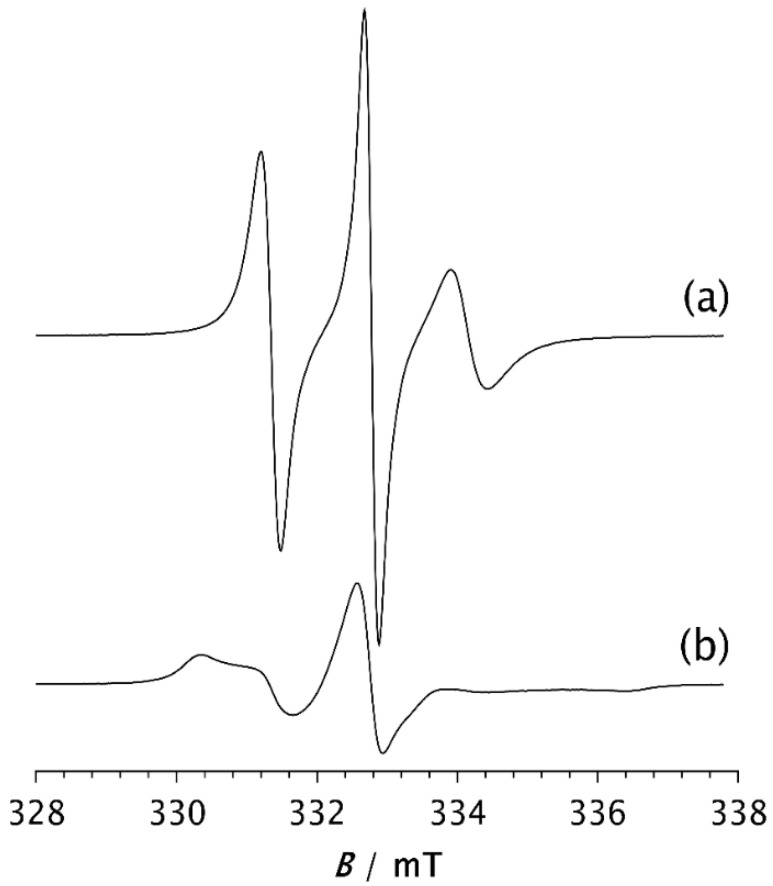
ESR spectra of the spin probe measured as a function of time for PDMS held at −85 °C for (**a**) 2 min and (**b**) 15 min, the time required to reach steady state (*t*).

To compare the dynamic behaviour of the composite samples with each other and with pristine PDMS, a temperature of −85 °C was selected as the most suitable for observing how the spectral shapes and the fractions of slow and fast components change over time after the samples are quenched to this temperature. Spectra of the spin probe in pristine PDMS, measured after holding the sample at −85 °C for 2 min and 15 min, are shown in Figure 4a and Figure 4b, respectively. Table 2 shows the intensity ratio of the broad (*I*_b_) and narrow (*I*_n_) components of the low-field lines, the fractions of the slow (*ϕ*_s_) and fast (*ϕ*_f_) components, the corresponding correlation times for the slow (*τ*_Rs_) and fast (*τ*_Rf_) components, and the time required to reach steady state (*t*). The strong difference in the shape of these two spectra may be related to the isothermal crystallisation kinetics. After 2 min, a relatively small number of crystals have formed, as shown in Figure 3a, and the segmental dynamics remains mainly fast, characterised by an average *τ*_R_ value of 2.59 ns, as shown in Table 2a. The spectrum recorded after 15 min remains unchanged with increasing time, suggesting that the crystallisation process is complete. This broad spectrum indicates that the fast segmental motions in the amorphous phase have been suppressed due to the restrictions imposed by the formation of the crystalline phase. In other words, almost the entire MAF region has transformed into the RAF, and the sample exhibits motion characteristic of polymer matrices at temperatures below *T*_g_. Such motional behaviour in semicrystalline PDMS was previously observed with high-field ESR [15,16]. Figure 3 schematically illustrates the structure of the semicrystalline matrix and the distribution of the spin probe within the MAF and RAF at steady state, reached at −85 °C.

**Figure 5:**
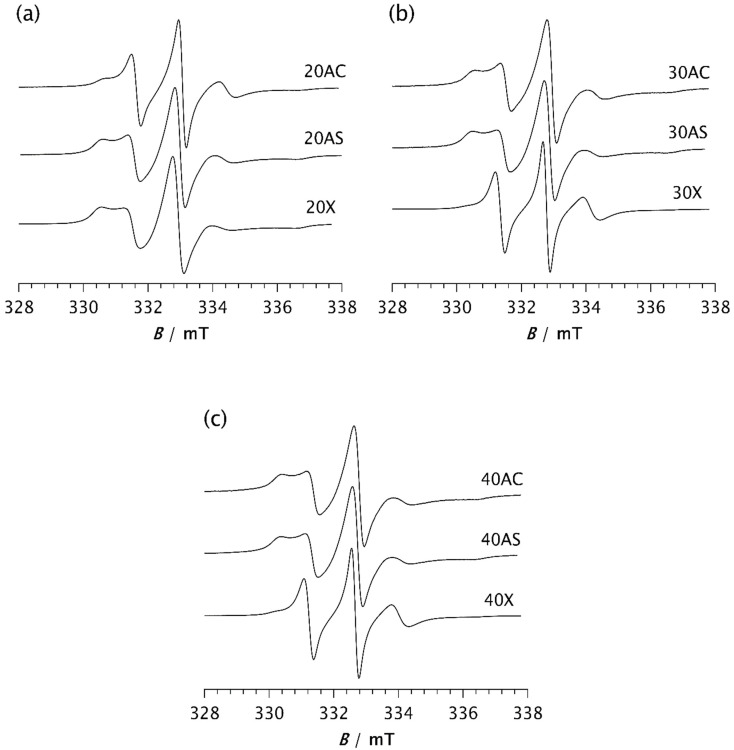
ESR spectra of the spin probe in PDMS composites: (**a**) 20AC, 20AS, and 20X, (**b**) 30AC, 30AS, and 30X, and (**c**) 40AC, 40AS, and 40X. The spectra were recorded 2 min after the samples reached −85 °C.

To investigate the influence of zeolite on the isothermal crystallisation process and molecular dynamics, the same measurements were performed on composite samples. Figure 5 shows the spectra of composite samples held at −85 °C for 2 min. A clear difference can be observed between the spectrum of pristine PDMS, as shown in Figure 4a, and those of the composite samples. The spectrum of PDMS does not clearly indicate the presence of two distinct types of motion, suggesting that PDMS remains predominantly in an amorphous phase after 2 min at −85 °C, characterised by a broad distribution of correlation times. However, compared to the spectrum recorded at 20 °C, an increase in the intensity of the central line and a slight line broadening are observed, indicating the onset of modest restriction in the local segmental motions due to the formation of a crystalline phase and the gradual emergence of the RAF.

In contrast, the spectra of the composites clearly show two well-resolved components, reflecting the dynamic inhomogeneity of the system associated with the presence of two distinct types of motion. One of the simplest parameters used to characterise the motional behaviour of the system is the intensity ratio between the broad and narrow components, *I*_b_/*I*_n_, as shown in Table 2a. Depending on the type and loading of the zeolite, this ratio ranges from 0.110 for samples 30X and 40X to 1.059 for sample 20X. These values are in good agreement with those obtained from computer simulations of the spectra, with the slow component fraction being the lowest for samples 30X and 40X at 36.5% and 43.3%, respectively. Samples 40AC and 40AS exhibit the highest fraction of the slow component, at 84.0% and 83.7%, respectively. A very similar value is observed for sample 20X, which shows a slow component fraction of 83.2%. However, the fraction of the slow component decreases significantly with increasing Zeolite X content, as shown in Table 2a.

The correlation times of the slow component are comparable for all composites, averaging around 11 ns, while the correlation times of the fast component vary slightly, ranging from 1.42 ns to 1.87 ns. In contrast to Zeolite X, Zeolite A in both its cubic and spherical forms shows a slight increase in the fraction of the slow component with increasing loading: from 62.2% to 84.0% for the cubic form and from 81.2% to 83.7% for the spherical form. This suggests an increased crystallisation rate at higher loadings. However, for the spherical form of Zeolite A, the effect of loading is less pronounced.

These results clearly demonstrate that adding zeolite to the PDMS matrix strongly promotes the crystallisation process, significantly increasing its rate. Similar effects on PDMS crystallisation have previously been reported in the presence of silica [7,8,37,38]. It has also been shown that the complexity of stress-induced crystallisation in PDMS is influenced not only by temperature and strain, but also by filler content [37]. In unfilled copolymer systems, crystallisation is initiated more slowly, likely due to the absence of heterogeneous nucleation sites provided by the filler surfaces [38]. Our results are consistent with these findings. However, at higher Zeolite X loadings (samples 30X and 40X), this effect is significantly reduced, resulting in a much lower fraction of the slow component compared to sample 20X, Figure 5 and Table 2a. This observation suggests that crystallisation proceeds more slowly during the initial stage. A similar trend at higher filler concentrations has also been observed by Ebengou and Cohen-Addad in PDMS–silica composites [9]. They showed that nucleation effects dominate at low silica contents, accelerating the crystallisation kinetics. In contrast, at high silica concentrations, the filler particles impose spatial constraints on crystal growth, leading to a slowdown in crystallisation kinetics. It was also found that composite samples with very high silica volume fractions (>45%) do not exhibit cold crystallisation. Studies on Zeolite X-based polymer composites have shown that particle agglomeration may occur at higher zeolite concentrations, when the filler reacts more favourably with itself rather than with the polymer [37]. It can therefore be assumed that another possible cause for this deviation in the Zeolite X composites, compared to the other samples, is the agglomeration of particles, resulting in a reduced interactive surface area, part of which provides heterogeneous nucleation sites. Consequently, the time required for the system to reach a steady state (*t*) is longer for samples 30X and 40X, at 7 and 13 min, respectively, as shown in Table 2b. It is clear that the time *t* for sample 40X is very close to that of pristine PDMS (15 min), while it is reduced to 3–5 min for the other samples. For the AC composites, increasing the zeolite content decreases the time *t* from 5 min for sample 20AC to 3 min for sample 40AC, indicating a slight increase in the crystallisation rate with higher zeolite content. In the case of AS composites, *t* varies only slightly from 4 min for samples 20AS and 30AS to 3 min for sample 40AS. The lower *t* value for sample 20AS compared to 20AC may be attributed to its larger specific surface area, caused by surface roughness [29]. This difference in specific surface area may also explain the significantly higher proportion of the slow component observed in sample 20AS after 2 min at −85 °C, compared to sample 20AC, as previously indicated.

From the perspective of the ESR-spin probe method, the time *t* can be related to the completion of crystallisation, although the literature data obtained by different methods for silica-filled PDMS report much longer times, sometimes exceeding 2 h [38]. This supports the hypothesis that zeolites may serve as more effective fillers for PDMS than silica. Considering the *t* values discussed above, it can be assumed that higher contents of Zeolite X cause mechanical hindrance during crystal formation, resulting in a moderate-to-low influence on the crystallisation rate. In contrast, the highest loadings of Zeolites AC and AS strongly increase the crystallisation rate.

**Figure 6:**
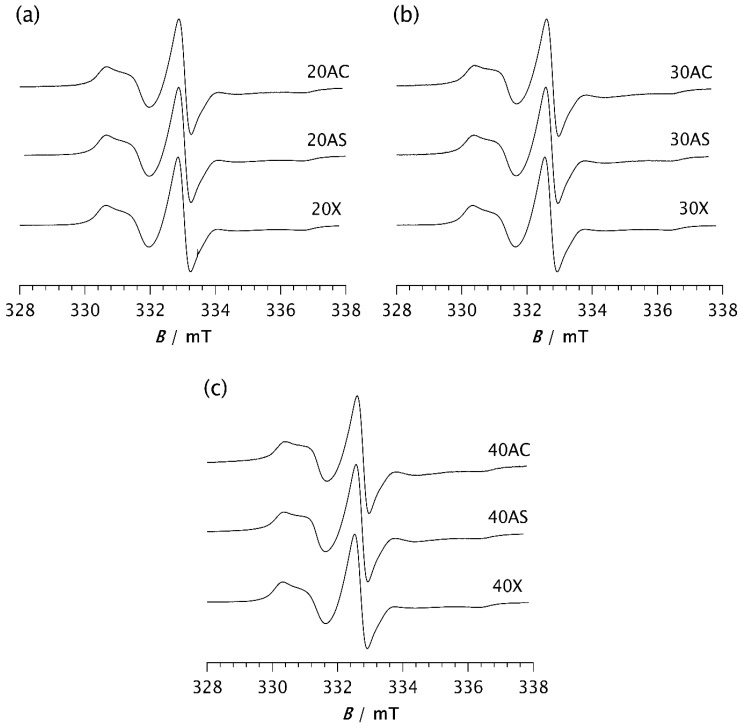
ESR spectra of the spin probe in PDMS composites: (**a**) 20AC, 20AS, and 20X; (**b**) 30AC, 30AS, and 30X; (**c**) 40AC, 40AS, and 40X. The spectra were recorded at −85 °C after the time *t* required to reach the steady state.

Once a steady state is reached, the spectra of all investigated samples are very similar, as shown in Figure 6. Computer simulations show only marginal differences in correlation times, proportions of slow and fast motions, and intensity ratios of the broad and narrow components, as seen in Table 2b. The fractions of the slow component are approximately the same, varying from 88.7% (30AS) to 91.6% (40AS). These values are very close to those of pristine PDMS (88.2%), as seen in Table 2b. The same applies to the ratios of the broad and narrow components, with only minor deviations. The *τ*_Rf_ values are somewhat higher than those calculated from the spectra recorded after 2 min, ranging from 1.85 ns for 20AS to 3.26 ns and 3.28 ns for 40AC and 30AC, respectively. In contrast, the *τ*_Rs_ values remain almost unchanged compared to those measured after 2 min and fluctuate slightly around 10–11 ns. A small increase in *τ*_Rf_ may indicate a stronger interaction between the matrix chain segments and the filler particles for the cubic form of Zeolite A. However, as indicated above, there are significant differences in the time *t* required to reach the steady state. This result strongly confirms the previously discussed influence of zeolites on the rate of isothermal crystallisation.

Considering the very similar fractions of the slow and fast components, and especially the closely matching *τ*_Rs_ values, no difference in dynamic behaviour within the RAF is observed across all studied samples once the steady state is reached, regardless of differences in the degree of crystallinity determined by DSC. Nevertheless, a slight increase in *τ*_Rf_ values at higher loadings, most pronounced for samples 30AC and 40AC, indicates a slowdown of dynamics in the MAF. Since this effect is less pronounced for Zeolite AS, and particularly for the Zeolite X composites regardless of loading, it can be concluded that zeolite morphology has a greater influence on molecular motion in the MAF region than the zeolite content itself.

### 3.2. DSC Analysis

**Figure 7:**
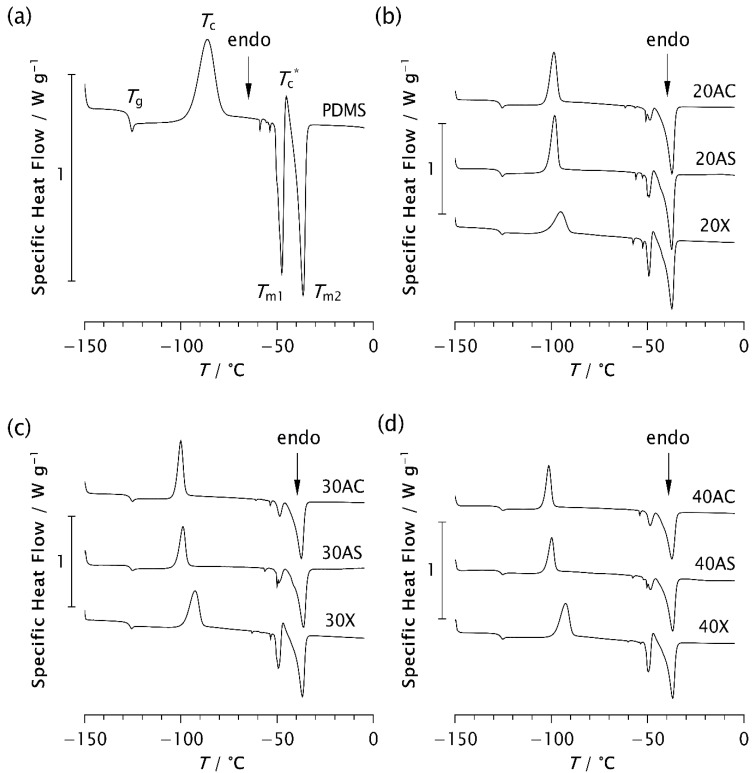
DSC thermograms of (**a**) pristine PDMS and PDMS–zeolite composites with the following loadings: (**b**) 20%, (**c**) 30%, and (**d**) 40%.

The results of the thermal analysis performed using DSC are shown in Figure 7 and Table 3, Table 4 and Table 5. All samples analysed exhibit glass transition at approximately −126 °C (−126.27 ± 0.18 °C), regardless of the zeolite type or content. This confirms that the addition of zeolites to PDMS does not affect *T*_g_. Some studies have also shown that the glass transition temperature remains unchanged despite variations in molecular weight, prior cooling rate, filler content, and degree of crosslinking [7,11,17]. An exothermic peak appears in the range from −101.3 °C to −86.15 °C, indicating cold crystallisation of the samples [4,7,8,38]. Its position on the temperature scale, *T*_c_, intensity, and width, depend on the type and content of the zeolite. In pristine PDMS, the peak appears at the highest temperature (−86.15 °C), while in all composite samples it is shifted to lower temperatures, as shown in Table 3. This finding is consistent with the previously observed temperature shift in the presence of silica [7,11]. Furthermore, it has been shown that the cold crystallisation temperature, as well as the area of the exothermic peak, depends not only on the filler content, but also on other parameters affecting the ability of the chains to crystallise during cooling.

Analysis of the *T*_c_ values shows that *T*_c_ decreases most significantly with Zeolite AC, followed by AS, while the effect is least pronounced with Zeolite X, as evident from Table 3. A similar trend is observed in the thermograms in Figure 7. For example, as shown in Figure 7c, the exothermic peak of sample 30X exhibits a smaller shift towards lower temperatures compared to samples 30AC and 30AS. Narrower and sharper peaks in the composites indicate a higher crystallisation rate compared to pristine PDMS. These results are consistent with the ESR data and suggest that zeolite morphology plays a more significant role in controlling the kinetics of isothermal crystallisation than zeolite content alone.

According to the literature, the large cold crystallisation peak is attributed to further crystallisation of imperfect crystals formed during the previous cooling stage [7]. This peak is reduced at lower cooling rates due to the formation of more perfect crystals. However, in this study, the cooling rate was kept constant for all samples. Therefore, the observed decrease in peak intensity can be attributed to the presence of zeolites, which may enhance the structural regularity of the resulting crystalline phase. A comparison of the enthalpy values Δ*H*_c_ in Table 4 shows that the composites exhibit lower values than pristine PDMS. Furthermore, Δ*H*_c_ decreases with increasing zeolite content in the AC and AS composites, whereas an increase in Δ*H*_c_ is observed for Zeolite X at higher concentrations.

In the temperature range between −50 °C and −36 °C, two endothermic peaks consistently appear at temperatures *T*_m1_ and *T*_m2_. In some samples, a small additional exothermic peak is observed between these two, at the temperature *T*_c_^*^, as shown in Figure 7 and Table 3. It is assumed that *T*_m1_ and *T*_m2_ correspond to the melting of less perfect and more perfect crystals, respectively, which form during the cooling and recrystallisation of metastable phases [4,7]. However, it may also be assumed that the temperatures *T*_m1_ and *T*_m2_ are related to differences in lamellar thickness, possibly corresponding to the melting of thinner and thicker lamellar crystals, respectively. The peak at *T*_c_^*^ is attributed to the recrystallisation of a portion of the crystals melted at *T*_m1_. As shown in Table 3, the values of *T*_m2_ are approximately the same for all samples, around −36 °C, while *T*_m1_ varies slightly from −47.66 °C for pristine PDMS to −50.06 °C for 30AS. A slight shift to a lower temperature may indicate that the crystal structures are somewhat less perfect, possibly due to the roughness of the zeolite surface. In contrast, an upward shift in *T*_m1_ was previously observed in PDMS as a result of more perfect crystal formation at a reduced cooling rate [7]. It should be noted that the high surface roughness of Zeolite AS results in a specific surface area that is 2.7-times larger than that of the Zeolite AC [29].

A clear decrease in the intensity of the endothermic peak at *T*_m1_, accompanied by significantly lower Δ*H*_m1_ values, is observed when zeolite is added to PDMS. At the same time, an increase in the second endothermic peak at *T*_m2_, along with the corresponding enthalpy values, is evident in the composite samples. This effect is somewhat less pronounced in the Zeolite X composites, where an additional small exothermic peak also appears at *T*_c_*. A similar but nearly negligible peak is observed in the 20AS sample, while it is absent in the other composites. These results support the assumption that the presence of zeolite influences not only the crystallisation rate, as detected by ESR, but also the crystalline structure, particularly its degree of perfection. Additional structural analysis is planned to confirm this assumption.

Table 5 presents the degree of crystallinity values calculated from the measured enthalpies for each phase transition observed during heating. These values provide valuable insight into the thermal behaviour of the samples and the extent of crystalline phase formation. Comparing these data across different composites clarifies how the degree of crystallinity depends not only on the zeolite content but also on the morphology of the zeolite particles. The overall degree of crystallinity (*χ*) was calculated using the following equation:

*χ* = *χ*_m1_ + *χ*_m2_ (2)

where *χ*_m1_ and *χ*_m2_ are the fractions of crystals melted at *T*_m1_ and *T*_m2_, respectively. The fraction of crystals formed during heating (*χ’*) was estimated as follows:

*χ’* = *χ*_c_ + *χ*_c_*. (3)

*χ*_c_ and *χ*_c_* represent the degree of crystallinity due to cold crystallisation and recrystallisation, respectively. The amount of crystals formed during rapid cooling (Δ*χ*) that did not recrystallise at *T*_c_ during heating was determined as follows:

Δ*χ* = *χ* − *χ*’. (4)

Sample 40AS is characterised by the lowest Δ*H*_c_ value, as shown in Table 4, and, consequently, the highest degree of crystallinity during cooling (Δ*χ* = 43.42%), as shown in Table 5. This can also be attributed to the high roughness of its outer surface [29], as discussed earlier. It is worth noting that only 16.56% of more perfect crystals are formed during rapid cooling of pristine PDMS.

The ESR measurements in this study were conducted at sub-zero temperatures (−85 °C), where PDMS exhibits semicrystalline behaviour. This temperature was chosen to investigate segmental dynamics within both the rigid amorphous fraction (RAF) and the mobile amorphous fraction (MAF), which are particularly relevant for understanding filler–matrix interactions in constrained environments. Uncrosslinked PDMS melt was used as a model system, as it remains liquid at room temperature and does not yield meaningful ESR data under ambient conditions due to the absence of segmental constraint. In contrast, the semicrystalline state at low temperatures enables detection of changes in molecular mobility influenced by zeolite fillers. These findings cannot be directly extrapolated to application-relevant conditions. However, using a model system provides fundamental insight into the effect of zeolite on molecular dynamics, which is an essential step before studying more complex systems.

## 4. Conclusions

The present observations highlight the sensitivity of the ESR-spin probe method for studying motional behaviour in the amorphous phase of semicrystalline PDMS and PDMS–zeolite composites. This information is essential for a better physical understanding of the properties of semicrystalline polymer systems. The results of this study show that incorporating zeolites into the PDMS matrix significantly affects both the kinetics and mechanisms of crystallisation, as well as the segmental dynamics of polymer chains. ESR spectroscopy revealed that the addition of zeolites increases the isothermal crystallisation rate. This effect strongly depends on both the type and morphology of the zeolite. At higher concentrations (30 wt% and 40 wt%) of Zeolite X, particle agglomeration may occur, reducing the interfacial area, and consequently, slowing crystallisation.

DSC analysis confirmed that the glass transition temperature remains unaffected by the addition of zeolites, while clear changes are observed in cold crystallisation and melting behaviour. Zeolite-containing composites exhibit a lower enthalpy of cold crystallisation (Δ*H*_c_ ≈ 13–21 J/g) and a shift in crystallisation and melting peaks, indicating increased structural regularity of the formed crystalline phase. These results are consistent with the ESR data and support the conclusion that zeolites act as heterogeneous nucleating agents, enhancing crystallisation and influencing the perfection of the resulting crystals.

Among all composites, the samples containing 30 wt% and 40 wt% of spherical Zeolite A showed the highest amounts of crystallites formed during rapid cooling, reaching 40.94% and 43.42%, respectively. This effect is attributed to the higher external surface roughness and larger specific surface area of the spherical Zeolite A particles. Overall, the study highlights that zeolite morphology has a greater impact than its content on both molecular mobility and crystallisation behaviour in PDMS composites. Future work should include detailed structural studies to further elucidate the relationship between the properties of the filler and the crystalline architecture of the polymer matrix.

## Figures and Tables

**Table 1:** Composition and labels of the investigated composite samples and pristine PDMS matrix.

Matrix	Zeolite Type	PDMS/wt%	Zeolite/wt%	Label
PDMS	-	100	0	PDMS
	A-cubic	80	20	20AC
	A-cubic	70	30	30AC
	A-cubic	60	40	40AC
	A-spherical	80	20	20AS
	A-spherical	70	30	30AS
	A-spherical	60	40	40AS
	X	80	20	20X
	X	70	30	30X
	X	60	40	40X

**Table 2:** Analysis of the ESR spectra measured at −85 °C (**a**) after 2 min and (**b**) after the time *t* required to reach the steady state. Shown are the intensity ratio of the broad (*I*_b_) and narrow (*I*_n_) components for the low-field lines, the fraction of the slow (*ϕ*_s_) and fast (*ϕ*_f_) components, and the corresponding correlation times of the slow (*τ*_Rs_) and fast (*τ*_Rf_) components.

(**a**)						
**Sample**	***I*_b_/*I*_n_**	***ϕ*_s_/%**		***ϕ*_f_/%**	***τ*_Rs_/ns**	***τ*_Rf_/ns**
PDMS	-	-		100.0	-	2.59
20AC	0.277	62.2		37.8	10.99	1.42
20AS	0.769	81.2		18.8	10.76	1.59
20X	1.059	83.2		16.8	10.39	1.87
30AC	0.710	77.0		23.0	10.89	1.49
30AS	0.940	83.5		16.5	10.78	1.59
30X	0.110	36.5		63.5	11.49	1.49
40AC	0.833	84.0		16.0	11.02	1.59
40AS	0.849	83.7		16.3	10.54	1.59
40X	0.110	43.3		56.7	10.51	1.49
(**b**)						
**Sample**	***I*_b_/*I*_n_**	***ϕ*_s_/%**	***ϕ*_f_/%**	***τ*_Rs_/ns**	***τ*_Rf_/ns**	***t*/min**
PDMS	1.667	88.2	11.8	11.74	1.87	15
20AC	1.769	90.3	9.7	11.12	1.86	5
20AS	1.754	90.4	9.6	11.01	1.85	4
20X	1.723	89.1	10.9	11.07	2.06	4
30AC	1.500	89.0	11.0	10.40	3.28	4
30AS	1.643	88.7	11.3	11.61	1.95	4
30X	1.778	88.8	11.2	11.18	2.04	7
40AC	1.531	89.0	11.0	10.27	3.26	3
40AS	1.571	91.6	8.4	9.68	2.67	3
40X	1.785	89.5	10.5	11.11	2.08	13

**Table 3:** Phase transition temperatures determined by DSC include glass transition temperature (*T*_g_), cold crystallisation temperature (*T*_c_), recrystallisation temperature (*T*_c_*), and melting temperatures (*T*_m1_ and *T*_m2_) for the investigated composites and pristine PDMS.

Sample	*T*_g_/°C	*T*_c_/°C	*T*_c_*/°C	*T*_m1_/°C	*T*_m2_/°C
PDMS	−126.35	−86.15	−45.18	−47.66	−36.53
20AC	−126.35	−98.62	-	−47.86	−37.37
30AC	−126.02	−99.94	-	−48.59	−37.38
40AC	−126.18	−101.3	-	−48.74	−37.32
20AS	−126.35	−98.13	−46.70	−49.25	−37.52
30AS	−126.01	−98.80	-	−50.06	−36.32
40AS	−126.18	−99.81	-	−48.58	−36.86
20X	−126.51	−95.16	−46.86	−49.43	−37.33
30X	−126.52	−92.64	−46.88	−49.49	−36.92
40X	−126.18	−92.65	−47.03	−49.60	−36.83

**Table 4:** Enthalpies of phase transitions for pristine PDMS and composite samples.

Sample	Δ*H*_c_/Jg^−1^	Δ*H*_c_*/Jg^−1^	Δ*H*_m1_/Jg^−1^	Δ*H*_m2_/Jg^−1^
PDMS	24.24	1.31	−12.60	−19.14
20AC	18.86	-	−3.46	−25.00
30AC	18.53	-	−3.24	−24.56
40AC	14.78	-	−3.57	−23.20
20AS	20.61	0.59	−3.93	−26.44
30AS	15.53	-	−4.10	−26.74
40AS	12.95	-	−3.87	−25.32
20X	14.04	0.71	−5.69	−23.64
30X	21.21	1.73	−8.20	−25.40
40X	20.77	1.77	−6.97	−23.75

**Table 5:** Degrees of crystallinity for the investigated samples, where *χ*_c_ and *χ*_c_* correspond to crystals formed during cold crystallisation and recrystallisation, respectively, and *χ*_m1_ and *χ*_m2_ correspond to crystals melted at *T*_m1_ and *T*_m2_, respectively. Δ*χ* is the crystalline fraction formed during rapid cooling.

Sample	*χ*_c_/%	*χ*_c_*/%	*χ*_m1_/%	*χ*_m2_/%	*χ’*/%	*χ*/%	Δ*χ*/%
PDMS	64.81	3.50	33.69	51.18	68.31	84.87	16.56
20AC	50.43	-	9.26	66.84	50.43	76.10	25.67
30AC	49.55	-	8.66	65.67	49.55	74.33	24.78
40AC	39.52	-	9.55	62.03	39.52	71.58	32.06
20AS	55.11	1.58	10.51	70.70	56.69	81.21	24.52
30AS	41.52	-	10.96	71.50	41.52	82.46	40.94
40AS	34.63	-	10.35	67.70	34.63	78.05	43.42
20X	37.54	1.90	15.21	63.21	39.44	78.42	38.98
30X	56.71	4.63	21.93	67.91	61.34	89.84	28.50
40X	55.53	4.73	18.64	63.50	60.26	82.14	21.88

## Data Availability

The original contributions presented in this study are included in the article/Supplementary Material. Further inquiries can be directed to the corresponding authors.

## Supplementary Materials

The following supporting information can be downloaded at https://www.mdpi.com/article/10.3390/polym17212911/s1, Figure S1: Morphological forms of the zeolites used: (a) cubic zeolite A, (b) spherical zeolite A, and (c) pyramidal zeolite X.
