# Supplementary material for: The Effect of Zeolite Morphology and Loading on the Local Segmental Dynamics and Crystallisation Behaviour of PDMS–Zeolite Composites
Source: Polymers (Basel). 2025 Oct 31;17(21):2911. doi: 10.3390/polym17212911 (PMC12609480; doi:10.3390/polym17212911)
Supplement: Supplementary file 1 [file polymers-17-02911-s001.zip › polymers-3930912-supplementary.pdf]

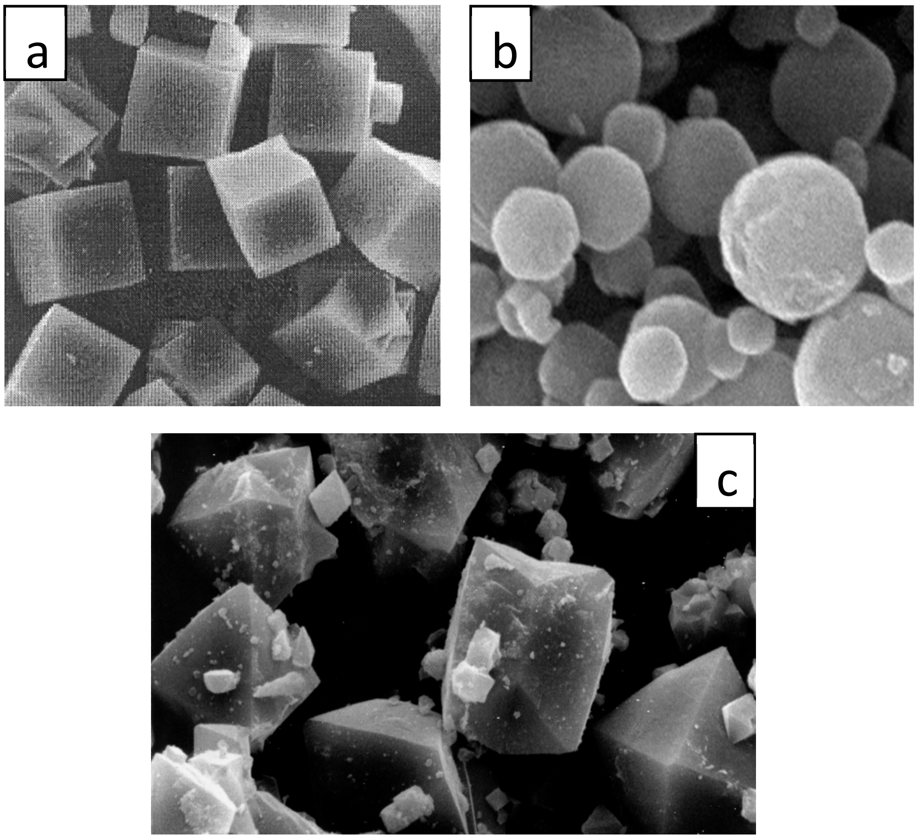

Figure S1. Morphological forms of the zeolites used: (a) cubic zeolite A, (b) spherical zeolite A, and (c) pyramidal zeolite X.
